# MIEN1 is tightly regulated by SINE Alu methylation in its promoter

**DOI:** 10.18632/oncotarget.11675

**Published:** 2016-08-29

**Authors:** Smrithi Rajendiran, Lee D. Gibbs, Timothy Van Treuren, David L. Klinkebiel, Jamboor K. Vishwanatha

**Affiliations:** ^1^ Department of Molecular and Medical Genetics, Institute for Cancer Research and Texas Center for Health Disparities, University of North Texas Health Science Center, Fort Worth, 76107, TX, USA; ^2^ Department of Biochemistry and Molecular Biology, University of Nebraska Medical Center, Omaha, 68198, NE, USA

**Keywords:** MIEN1, prostate, DNA methylation, epigenetic regulation, SINE Alu

## Abstract

Migration and invasion enhancer 1 (MIEN1) is a novel gene involved in prostate cancer progression by enhancing prostate cancer cell migration and invasion. DNA methylation, an important epigenetic regulation, is one of the most widely altered mechanisms in prostate cancer. This phenomenon frames the basis to study the DNA methylation patterns in the promoter region of MIEN1. Bisulfite pyrosequencing demonstrates the MIEN1 promoter contains a short interspersed nuclear Alu element (SINE Alu) repeat sequence. Validation of methylation inhibition on MIEN1 was performed using nucleoside analogs and non-nucleoside inhibitors and resulted in an increase in both MIEN1 RNA and protein in normal cells. MIEN1 mRNA and protein increases upon inhibition of individual DNA methyltransferases using RNA interference technologies. Furthermore, dual luciferase reporter assays, in silico analysis, and chromatin immunoprecipitation assays identified a sequence upstream of the transcription start site that has a site for binding of the USF transcription factors. These results suggest the MIEN1 promoter has a SINE Alu region that is hypermethylated in normal cells leading to repression of the gene. In cancer, the hypomethylation of a part of this repeat, in addition to the binding of USF, results in MIEN1 expression.

## INTRODUCTION

Epigenetic regulation of genes involves non-genetic modifications of DNA and/or histones. Such regulation leads to the transcriptional activation or repression, thereby maintaining an accurate spatial-temporal expression pattern, culminating in cellular homeostasis [1]. Subsequently, the deregulation of the epigenetic mechanisms results in aberrant gene expression. DNA methylation, an important epigenetic modification, is often deregulated in various cancers [2–4]. Usually, a global hypomethylation of repeat sequences including interspersed non-coding regions, accompanied by gene-specific DNA hypermethylation in the promoters of the tumor suppressor genes are detected in many cancers [4–7]. With the developments in the field of genomics, including next generation sequencing, many genes that are altered in various cancers have been identified. Among these genes, a vast majority that are down regulated in cancers correlate to genes exhibiting hypermethylated promoters, thus corroborating the previous observations. These studies, along with some others in the past, allude to the potential use of the methylation pattern signatures as biomarkers for early detection of cancer [8–12].

Prostate cancer, next only to lung cancer in terms of cancer related deaths, is estimated to account for 27,540 deaths in 2015 [13]. Mortality in prostate cancer is the result of metastasis of the cancer, a complex process involving several players. Many genes that are involved in apoptosis, cell cycle regulation, and hormone regulation (apart from genes that act as tumor suppressors by inhibiting oncogenic processes) have been shown to be hypermethylated in prostate cancer [3, 7, 10, 11, 14, 15]. Although most of the focus has been in terms of DNA hypermethylation, it is important to also consider the hypomethylation patterns. These patterns, directly or through repeat elements, drive the expression of oncogenes, resulting in genomic instability during tumor progression [4, 6, 12, 16–19]. Therefore, generalized demethylation may not be the most effective approach to when considering efficient treatment options. Clarifying the different genes involved in the tumor progression will create novel avenues in developing impactful methylation based targeting strategies.

Studies previously conducted by our lab and other groups have identified migration and invasion enhancer 1 (MIEN1) as an important gene involved in cancer progression [20, 21]. MIEN1 is located in the 17q12 region of the human chromosome, alongside HER2/*neu*, a region of extreme importance in various cancers [22, 23]. This region has also been shown to be important with respect to a more aggressive form of prostate cancer, castration resistant prostate cancer. While increased MIEN1 facilitates tumor progression [24, 25, 26, 27], its expression is low to negligible in various normal cells and tissues, making MIEN1 an attractive biomarker and therapeutic target.

In the present study, we determined the involvement of epigenetic regulation of MIEN1 by DNA methylation of its putative promoter. Our study shows that the MIEN1 promoter has a SINE Alu region that is hypomethylated in cancer, resulting in an increased expression of MIEN1 in cancer [5, 28]. Inhibition of methylation in the immortalized normal epithelial cells by various methods including knocking down of the DNA methyltransferases led to an increase in MIEN1 transcript and protein. Additionally, chromatin immunoprecipitation assays revealed binding of a transcription factor that regulates the MIEN1 expression in conjunction with the methylated SINE Alu. Together, our results prove that MIEN1 promoter methylation is very important in repressing the gene in normal cells and that this regulation is lost in cancer.

## RESULTS

### MIEN1 putative promoter has DNA methylation responsive elements

**Figure 1:**
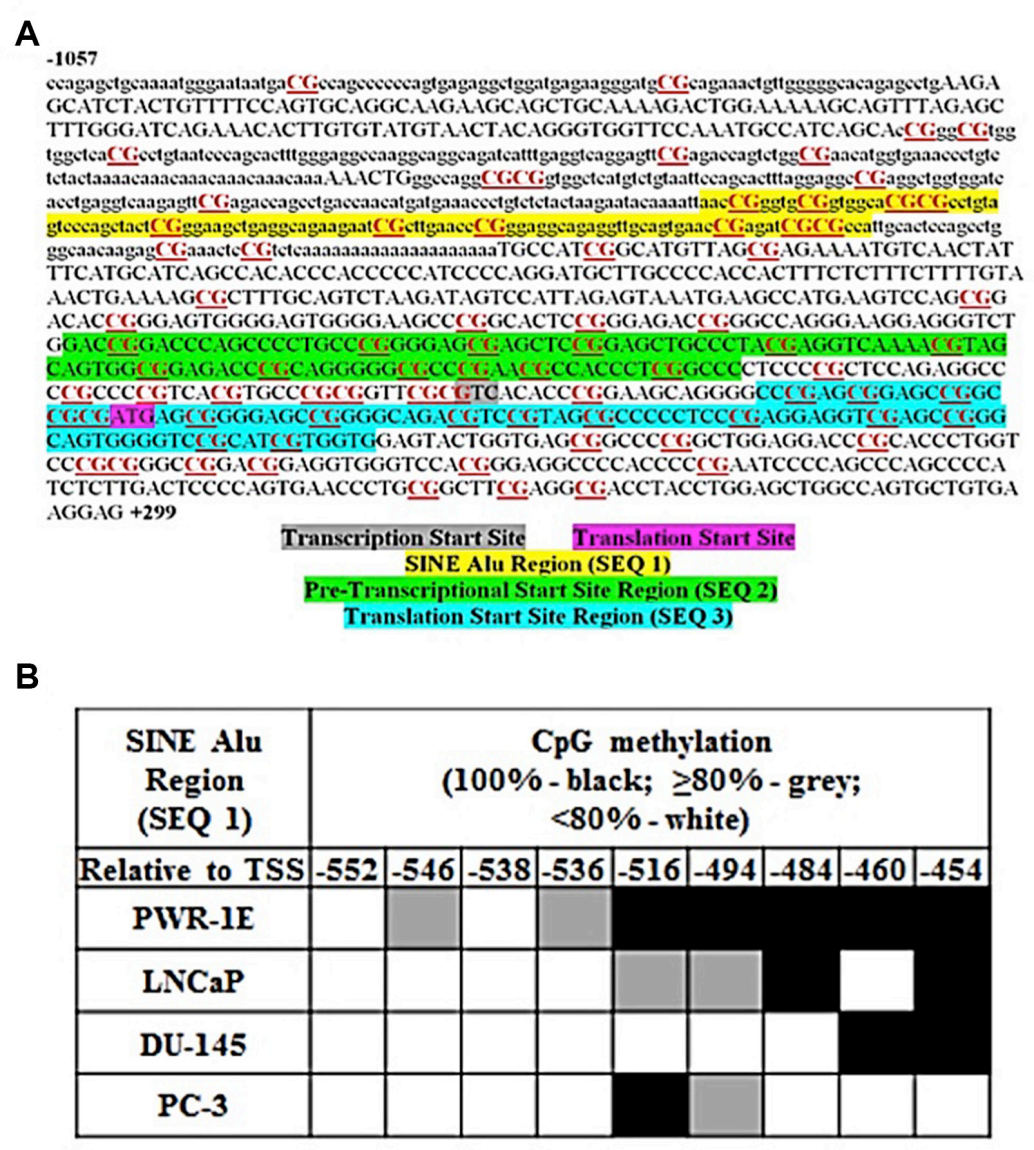
MIEN1 putative promoter region and potential methylation sites (**A**) MIEN1 promoter sequence from UCSC: CpG dyads and islands are underlined and in red; the SINE Alu repeat is represented in lower case. (**B**) Bisulfite sequencing based % methylation at the CpG sites within the SINE Alu region with respect to the technical control of DNA treated with SssI methyltransferase (100% methylation).

The sequence for MIEN1 putative promoter region was obtained from the UCSC Genome Browser and NCBI gene database. Upon scanning, we observed that this region contains numerous CpG dyads, CpG islands and a short interspersed nuclear element (SINE) Alu repeat. We determined the region to be interrogated for methylation within the MIEN1 gene that would possibly regulate the expression of MIEN1 by analyzing a previous study of high throughput methylation performed on normal and malignant ovarian epithelial and fallopian tube epithelial tissues (GSE81228) where we found the Alu region to be differentially expressed between normal and malignant tumors [29]. Using bisulfite pyrosequencing, we then examined three regions, namely, a portion of the SINE Alu region, a pre-transcription start site region and a translation start site region (Figure 1A), which all contained potential methylation CpG sites, in detail in immortalized normal prostate epithelial PWR-1E cells, androgen-dependent LNCaP cells and androgen-independent DU-145 and PC-3 cancer cells. Since SINE Alu repeats constitute 11% of the human genomic sequence, a bisulfite primer set (BSP, Supplementary Table S1) was initially used to extract the appropriate sequence containing the region of interest to us in its entirety. Subsequently, the pyrosequencing of the three-100bp regions were performed using the sequence specific primers (SEQ, Supplementary Table S1) to determine the % methylation at each of the CpG sites within that region. The sequencing data showed that the methylation was less than 30% at any given CpG site in both the pre-transcription start site as well as the translation start site regions across all the cell lines (Supplementary Figure S1). On the contrary, variation in the methylation pattern between the normal and cancer cells was observed in about half of the CpG sites located within the sequenced SINE Alu region (Figure 1B). Within this differentially methylated region, PWR-1E exhibited complete methylation in all the five sites, while two sites in LNCaP were 100% and 80% methylated. In contrast, DU-145 and PC-3 demonstrated methylation in only two sites totally. Together, these results demonstrate that the methylation of SINE Alu region in the MIEN1 putative promoter is definitely lost in cancer compared to normal cells, thus supporting the known phenomena of global hypomethylation in cancer.

### MIEN1 expression is altered upon pharmacological inhibition of DNA methylation

**Figure 2:**
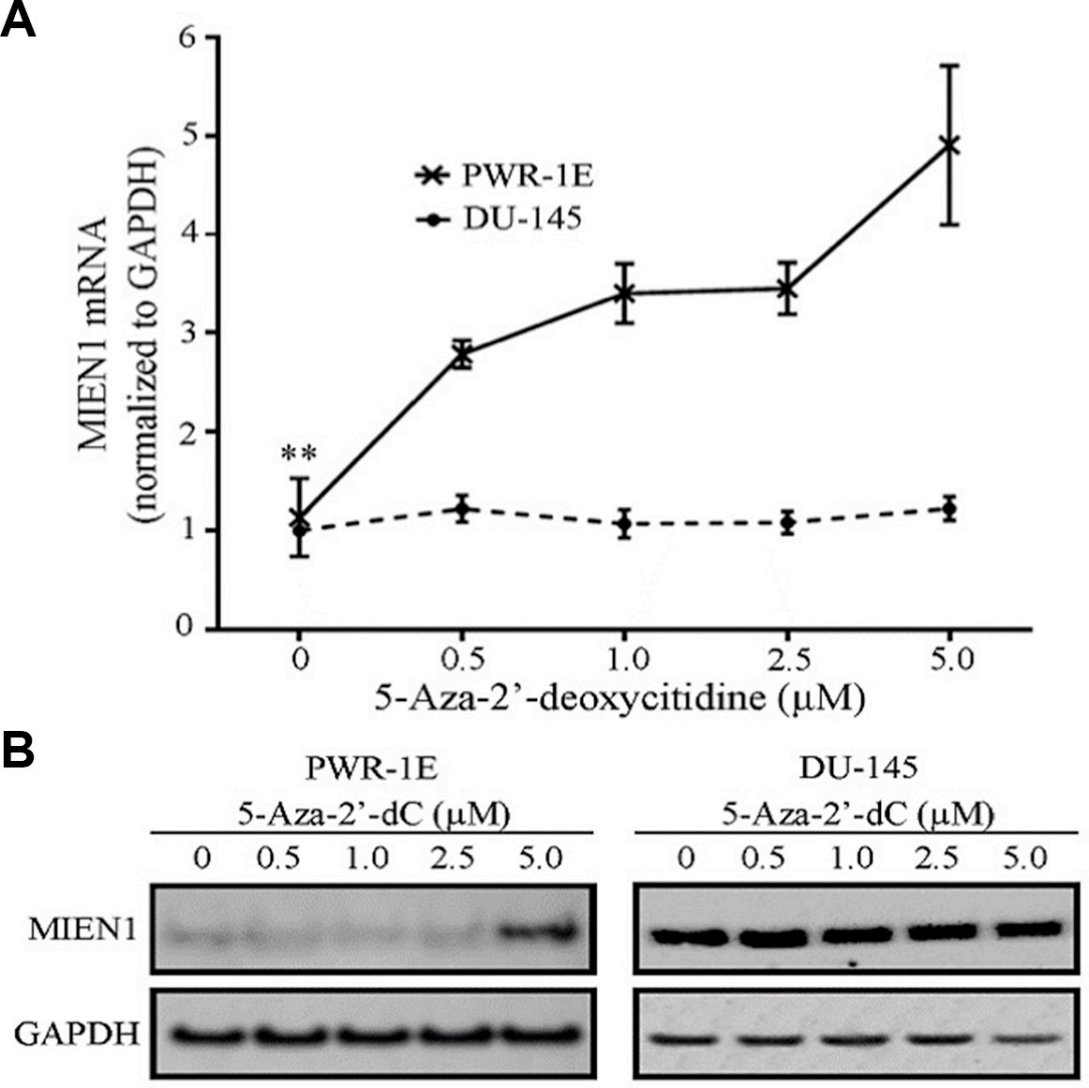
MIEN1 RNA and protein increases upon global methylation inhibition using 5-Aza-2′-deoxycytidine (**A**) qPCR showing the MIEN1 expression normalized to GAPDH (internal control) upon different concentrations of 5-Aza-2′-dC treatment in PWR-1E and DU-145 cells. (**B**) Western Blotting showing the MIEN1 expression upon 5-Aza-2′-dC treatment in PWR-1E and DU-145 cells; GAPDH was used for normalization. The *P*-values were computed using Student's *t-test* between the control and the indicated concentrations of 5-Aza-2′dC treatments. ***P ≤ 0.001, *P ≤ 0.05.

We next hypothesized that the loss of methylation of the SINE Alu repeat in the MIEN1 promoter potentially results in higher expression of MIEN1 in cancer compared to normal cells. To test this, we examined the effects of DNA demethylation on MIEN1 expression. PWR-1E and DU-145 cells were exposed to varying concentrations of the nucleoside analog and global inhibitor of DNA methylation, 5-Aza-2′-deoxycitidine (5-Aza-2′dC), for 72 hours. MIEN1 transcript and protein levels were assessed by qPCR and western blotting respectively. While an increase in MIEN1 RNA (Figure 2A) and protein (Figure 2B, left) were observed in PWR-1E cells treated with 5-Aza-2′dC compared to the vehicle, these levels (Figure 2A and 2B, right) remained unaltered in DU-145 cells, independent of the treatments. When we shortened the 5-Aza-2′-dC treatment to 48 hours, such that the cells would only undergo one replication cycle before collecting the RNA and protein, we observed the same patterns (Supplementary Figure S2). The expression patterns of MIEN1 in PC-3 cells were similar to the pattern observed in DU-145 (Supplementary Figure S2).

**Figure 3:**
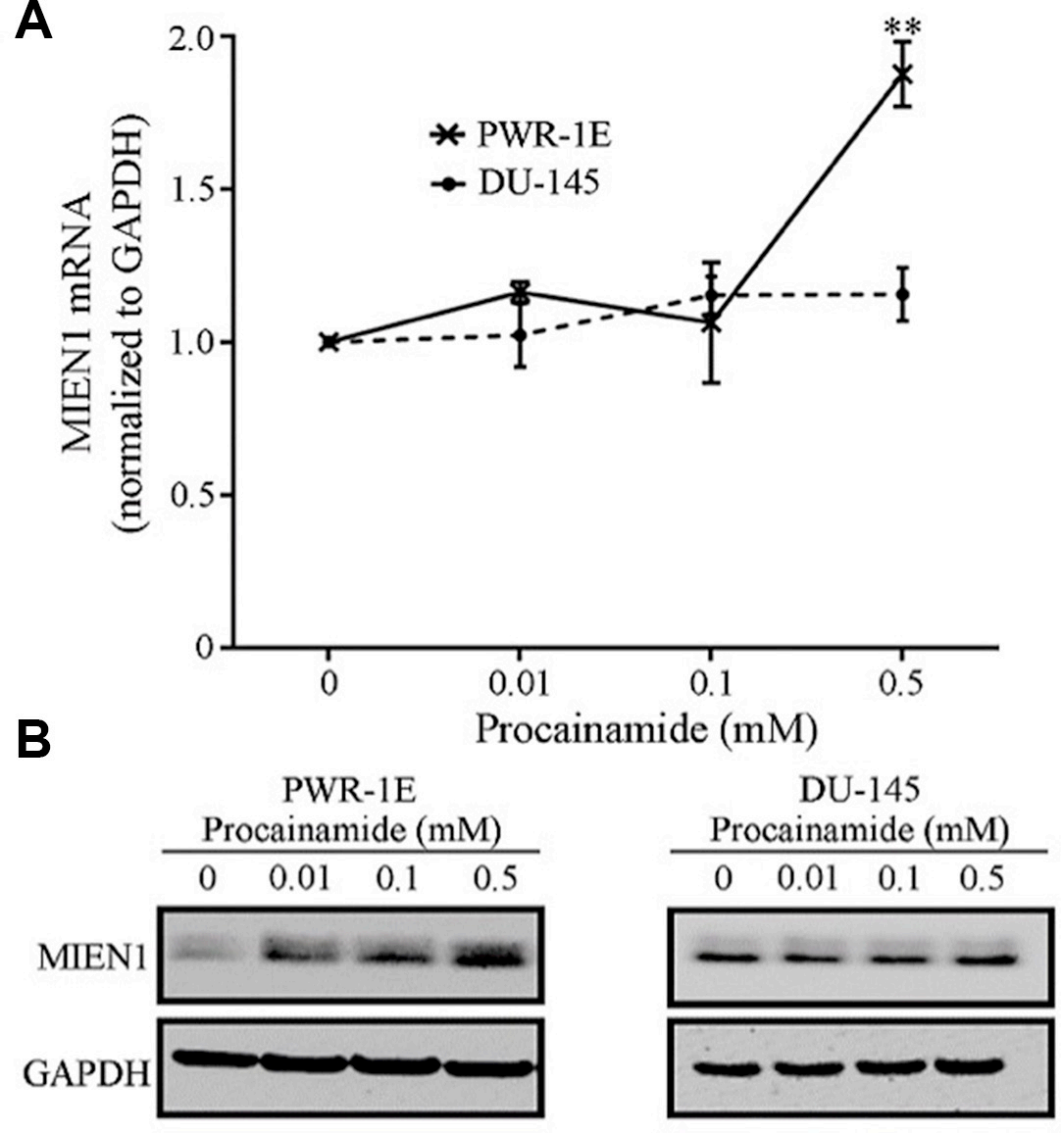
MIEN1 expression upon treatment with the non-nucleoside inhibitor, Procainamide MIEN1 expression normalized to GAPDH (internal control) upon different concentrations of Procainamide treatment in (**A**–**B**) PWR-1E and DU-145 cells, as depicted by (A) qPCR and (B) Western blotting. The *P*-values were computed using Student's *t-test* between the control and the various concentrations of procainamide. **P ≤ 0.01.

Next, to determine if the effects observed were a result of hindering the maintenance DNA methyltransferase, DNMT1, we treated PWR-1E and DU-145 cells with the pharmacological inhibitor of DNMT1, procainamide (PCN). MIEN1 expression was significantly induced at the transcriptional and protein levels after 96 hours of procainamide treatment in PWR-1E cells (Figure 3A and 3B). Similar to the effects obtained with the 5-Aza-2′dC treatment, no induction of MIEN1 was observed in DU-145 or PC-3 cells upon treatment with any concentration of procainamide (Figure 3A and 3B, Supplementary Figure S2). A closer look at the fold changes revealed that the MIEN1 mRNA was about 3- to 5-fold higher than the control upon 5-Aza-2′dC treatment (depending on the concentrations used), but with procainamide treatment, the maximum increase was ∼2-fold, thus indicating the possible role of both maintenance as well as *de novo* methyltransferases in the methylation of the putative MIEN1 promoter region.

### A combinatorial inhibition of DNMTs is necessary for the complete demethylation of MIEN1 promoter, resulting in MIEN1 expression

**Figure 4:**
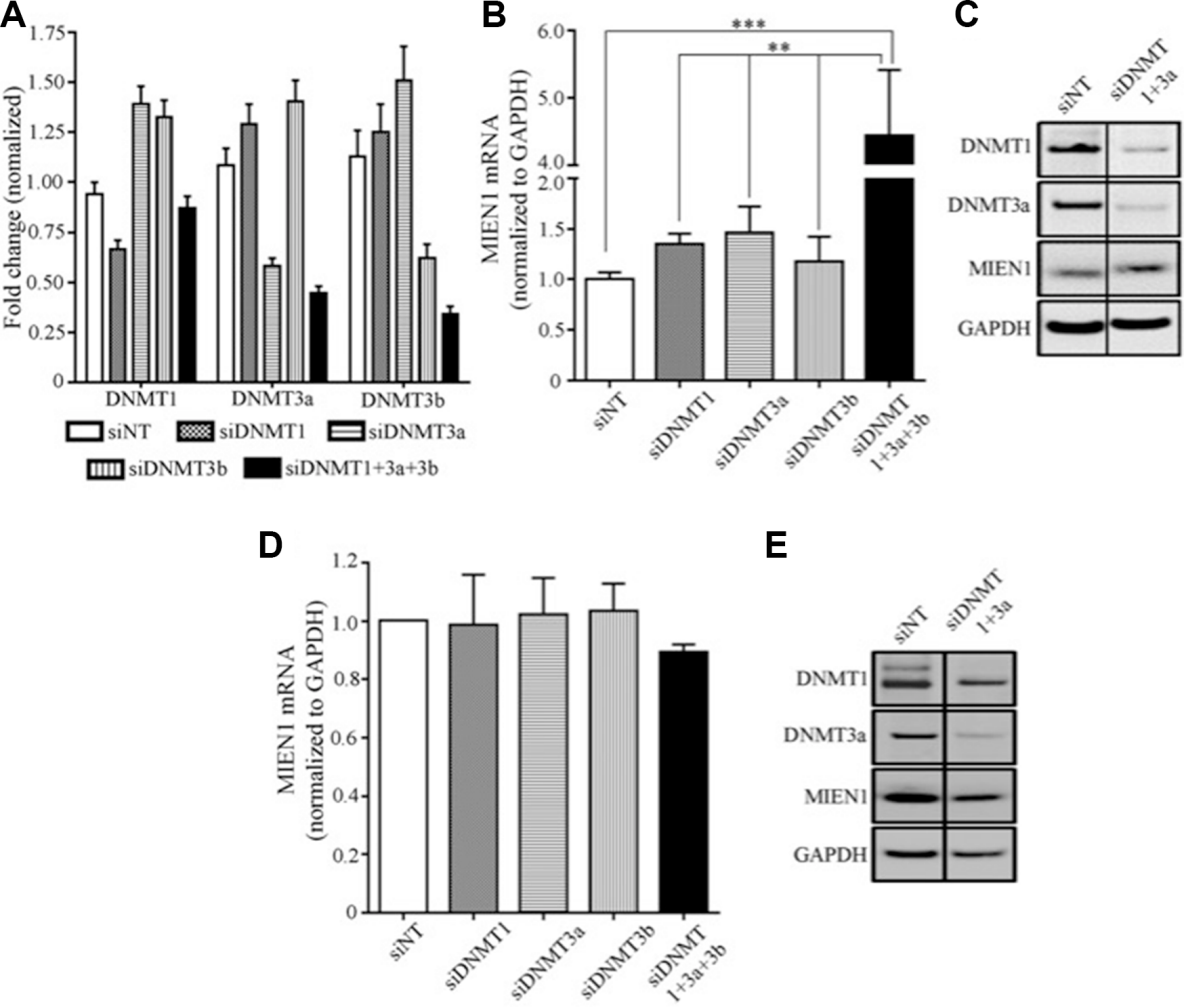
MIEN1 expression upon knockdown of DNA methyltransferases in PWR-1E (**A**–**C**) MIEN1 expression upon different DNMT knockdown in PWR-1E cells as shown by (A–B) qPCR and (**C**) Western Blotting. MIEN1 expression upon different DNMT knockdown in DU-145 cells as shown by (**D**) qPCR and (**E**) Western Blotting. The *P*-values were computed using One-way ANOVA to compare all the groups and then followed by Tukey's Post Hoc comparison to obtain the pairwise significances between the treatments. ***P ≤ 0.001; **P ≤ 0.01.

The use of pharmacological inhibitors is often accompanied by extreme cellular toxicity, not to mention the possibility to drive mutations. Hence, we next used RNA interference technology to determine the effects of each individual DNMT on MIEN1 expression. The PWR-1E cells were transfected with siRNA against DNMT1, DNMT3a, DNMT3b or the combination (DNMT1, DNMT3a and DNMT3b). GFP targeting siRNA was used as the non-targeting control. The qPCR analysis showed MIEN1 expression to be slightly elevated upon DNMT1 and DNMT3a knockdown (∼1.5-fold), though this was not significant (Figure 4B). On the other hand, silencing all the three DNMTs significantly increased MIEN1 mRNA (Figure 4B). The efficiency of the knockdown of DNMTs was determined by qPCR of the DNMTs at the same time that MIEN1 expression was tested (Figure 4A). To validate if the increase in mRNA did indeed result in an increase in the MIEN1 protein, total protein was isolated after PWR-1E cells were transfected with siRNA against the DNMTs. DNMT3b was not knocked down since no increase in MIEN1 mRNA was observed upon DNMT3b knockdown. Our results showed an increase in MIEN1 upon knocking down the DNMTs (Figure 4C), though the combined knockdown did not have any additive effect, unlike what was seen at the mRNA level. In DU-145, the knockdown of the DNMTs led to no alteration in MIEN1 mRNA or protein, as anticipated (Figure 4D and 4E). Taken together, these results imply that MIEN1 is indeed under the influence of methylation (DNMT1, DNMT3a and DNMT3b). In normal cells, the SINE Alu region in the MIEN1 promoter is methylated thus keeping the transcription of this gene under check; but in cancer, the hypomethylation results in transcriptional activation of the gene.

### Activity of MIEN1 promoter is influenced by SINE Alu

**Figure 5:**
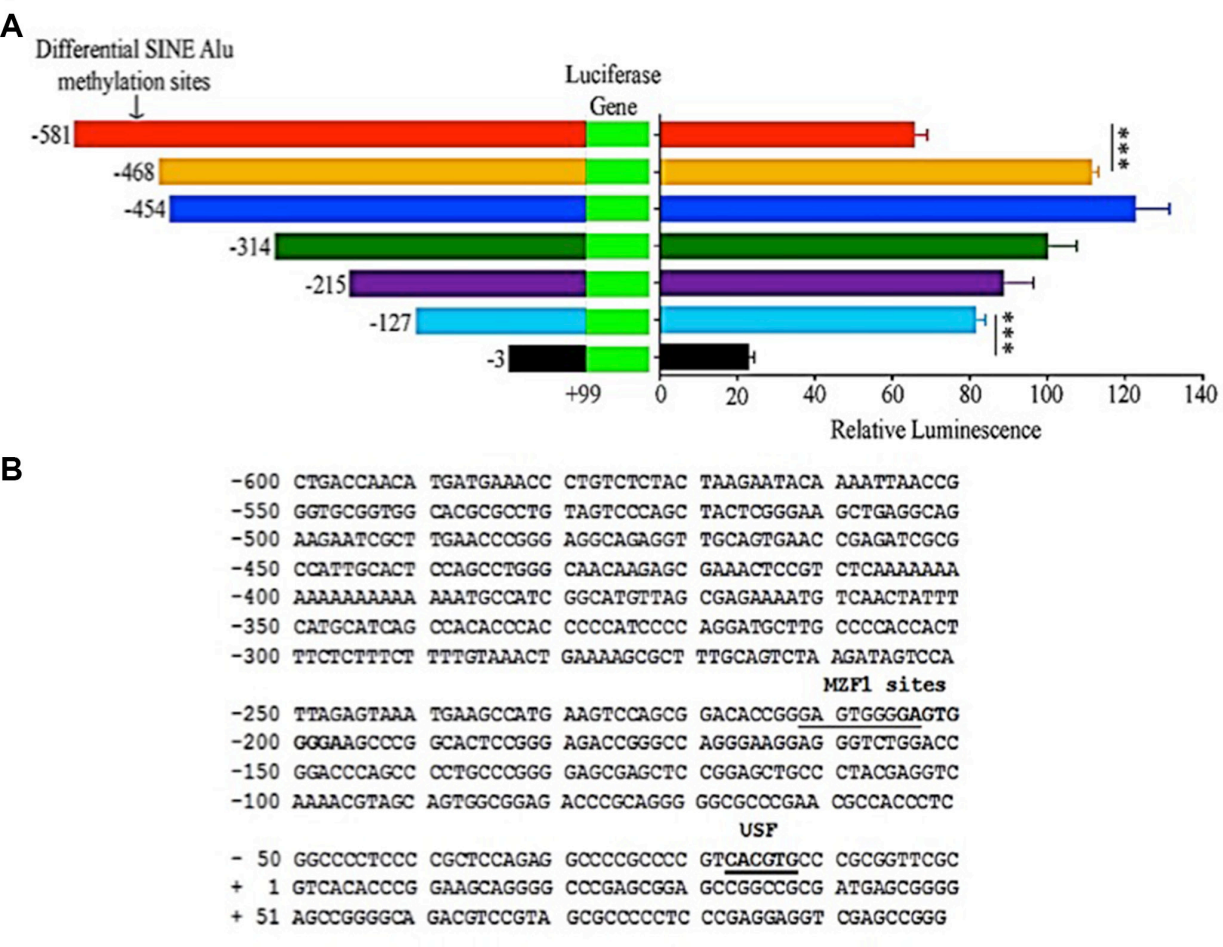
MIEN1 putative promoter activity as determined by luciferase assay and the potential transcription factor binding sites in the region (**A**) Cloning of various regions upstream of MIEN1 transcription start site into pGL3-basic vector. Relative luminescence (Firefly/Renilla ratio) obtained upon transfecting cells with the different pGL3 constructs. (**B**) The −600 to +99 region that contained the SINE Alu region and the proximal putative promoter of MIEN1 based on the known transcription start site (NCBI, UCSC) was used as the template, with a threshold set higher than default, in TFSEARCH software to obtain the putative transcription factor binding motifs in the region. The *P*-values were computed using Student's *t-test* between every two consecutive constructs. ***P ≤ 0.001.

Next, in order to determine the MIEN1 promoter activity, we cloned the different fragments of the putative MIEN1 promoter upstream of pGL3-Luciferase vector. We included various constructs of the promoter, either with or without the SINE Alu fragment we studied with bisulfite sequencing. The luciferase reporter assay confirmed our previous findings - the plasmids containing the SINE Alu region (−581/+99) exhibited significantly lower promoter activity compared to plasmids lacking at least some of the CpG sites within that SINE Alu segment (−468/+99, −454/+99 and −314/+99) in DU-145 (Figure 5A) and PWR-1E (Supplementary Figure S3) cells. Additionally, it is known that an interplay between various cellular mechanisms and gene repression by methylation exist. In order to identify elements in the proximity of the transcription start site that could potentially assist the epigenetic regulation by the methylation at the SINE Alu, we performed a bioinformatics search of that region using TFSEARCH [30]. The TFSEARCH analysis predicted binding sites for Myeloid Zinc Finger 1 (MZF1) and upstream stimulatory factor (USF) (Figure 5B). To further confirm if these potential regulatory sites are indeed important for the transcriptional regulation of MIEN1, we conducted luciferase reporter assays with and without the binding site(s) for these transcription factors. No significant difference in the activity was observed when the site for MZF1 was deleted; but the loss of the region containing the USF binding site (between −127 and −3) very significantly abrogated the transcriptional activity (Figure 5A and 5B).

### USF is a transcriptional activator of MIEN1

**Figure 6:**
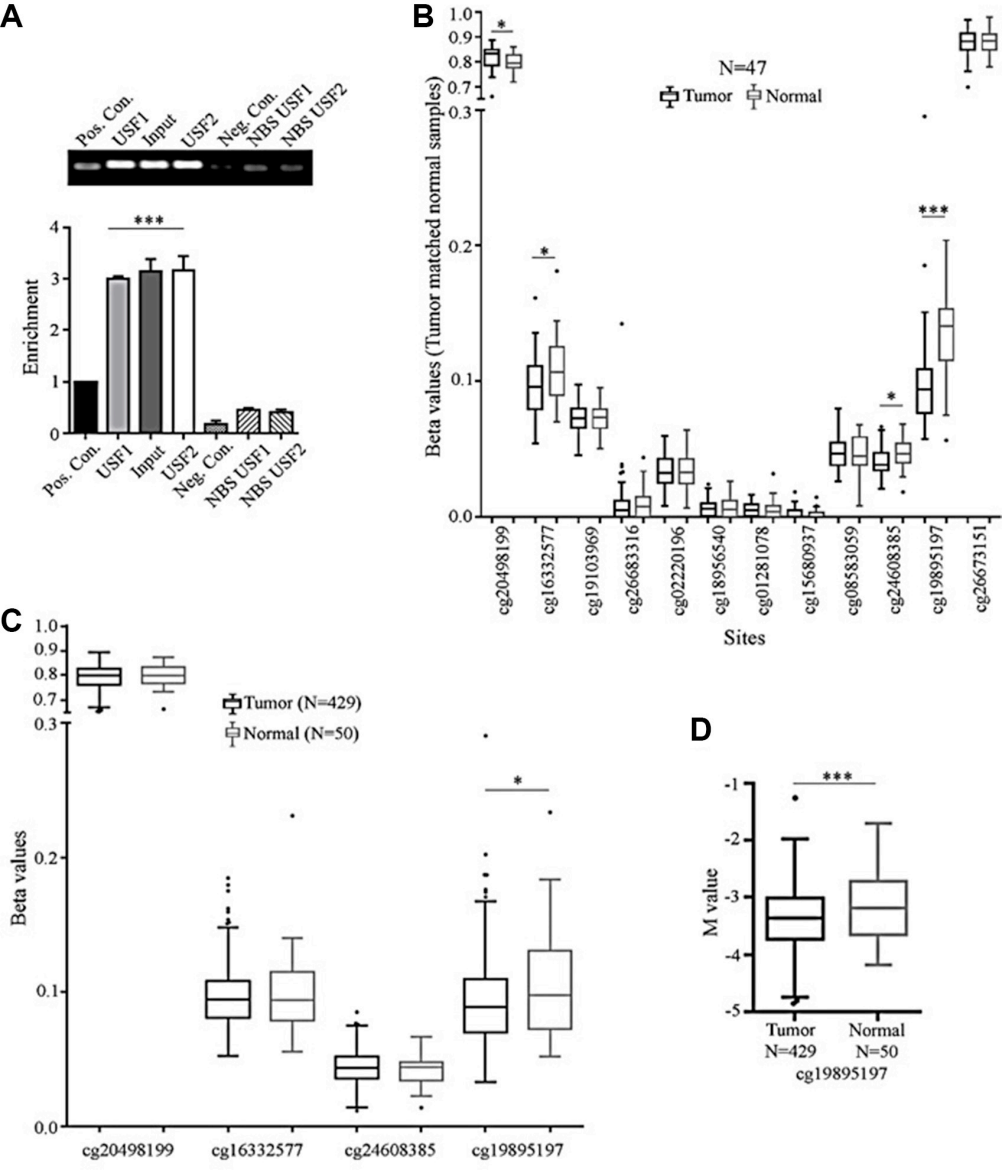
USF binds to MIEN1 promoter; while TCGA depicts cg sites that may be differentially methylated between tumor and normal tissues (**A**) ChIP analysis of USF1 and USF2 regulatory binding potential of the MIEN1 promoter. (**B**) TCGA analysis of the beta(β) values of MIEN1 methylations sites between tumor and matched normal prostate samples. (**C**) TCGA analysis of beta (β) MIEN1 methylation sites between prostate tumor samples and normal prostate samples. (**D**) TCGA analysis of M values, a robust measurement for methylation sites, prostate tumor and normal samples. The *P*-values were computed using Student's *t-test*. ***P ≤ 0.001, **P ≤ 0.01, *P ≤ 0.05.

Next, to confirm if the USF binding is a true and positive regulatory signal, we performed chromatin immunoprecipitation of the promoter region with USF1 and USF2 antibodies and amplified that region in the MIEN1 promoter by PCR. As seen in Figure 6A, both USF1 and USF2 bind to the putative MIEN1 promoter with almost equal efficiencies and result in ∼3-fold enrichment of the MIEN1 promoter region compared to the various negative controls (binding site PCR after negative antibody IP, non-binding site PCR after either USF antibody IPs). As a positive control, the same site was amplified from the input DNA (Input) or positive control antibody was used for IP.

### Significant difference is present in the extent of methylation at a specific site between prostate cancer and normal states in the putative MIEN1 promoter

It is well known that the methylation patterns between prostate cancer and normal states are significantly different globally [31]. Here, using existing information with the pre-designed TCGA methylation probe sets we analyzed the MIEN1 region for differences in methylation patterns. Initial comparison between tumor and matched normal showed four out of the twelve sites to be hypomethylated in cancer compared to the normal counterparts in a sample set of 47 patients (Figure 6B). To determine if this difference was truly a universal phenomenon observed between all prostate tumor and normal patients, we studied the methylation status, of only these four sites, in the entire TCGA prostate tumor database for methylation, using beta values. As seen in Figure 6C, upon increasing the sample size, the significant differences between the disease and non-disease states quickly disappear; leaving only one site, cg19895197, to still be significantly hypomethylated in cancer. To further validate this difference, we compared the M values, a more robust measurement, between these samples, instead of the beta values, and still retained significantly lower methylation status in the cancer compared to normal tissue at that site (Figure 6D), thereby confirming that the cg19895197 site was differentially methylated between normal and cancer.

## DISCUSSION

MIEN1, a novel gene in the 17q12 region of the human chromosome, is not only overexpressed in various solid tumors like oral and ovarian, but also confirmed as a prognosticator in breast cancer [24]. Our lab has previously demonstrated that MIEN1 enhances the migration and invasion of prostate cancer cells by activating the Akt/NF-κB pathway which leads to an increase in certain proteases and angiogenic factors, and by altering actin cytoskeleton structures (propelling cellular movement) [24, 32]. Given the importance of MIEN1 in various cancers, determining the regulator(s) that maintain its levels during disease-free homeostasis is essential to enable its use as a target. The microRNA-mediated regulation of MIEN1 proves that MIEN1 mRNA is prevented from forming a functional protein by hsa-miR-940 [33]. However, this does not explain nor does it negate the possibility of an inherently higher transcriptional activation of MIEN1 in cancer compared to normal cells. Thus, the current study aims to understand the pre-transcriptional as well as the transcriptional regulation of MIEN1.

In this study, the sequence analysis of the putative MIEN1 promoter region in immortalized normal epithelial cells and prostate cancer cells reveal a pattern of hypomethylation of the SINE Alu segment in cancer cells. This is in agreement with the existing literature suggesting SINE Alu regions to be commonly hypomethylated in cancers [34, 35]. The SINE Alu elements belong to a class of gene regulatory elements called retrotransposons [28, 36] that have been shown to become less mobile upon genetic regulation [6, 37]. A conducted genome wide study demonstrated that the SINEs enriched in cancers tend to be close to the transcriptional start sites of genes that are less methylated in cancer [5, 6]. This would imply the presence of this region (around 550 bases from the transcription start site of MIEN1) to be a strong indicator of lower methylation in cancer. In addition, of the three predicted regions, only the SINE Alu region showed distinction in terms of methylation between the cancer and normal cells. This phenomenon demonstrates the importance of such repeat elements in gene regulation. Furthermore, the luciferase reporter assays validated the transcriptional activity associated with the SINE Alu region.

Methylation inhibition can be achieved by various means [38]. In this study, both pharmacological inhibitors as well as RNA interference have been used to inhibit DNA methylation. The use of 5-Aza-2′dC as an epigenetic modulator in solid tumors has many associated complications (induction of mutations, reduction of stability and increased cytotoxicity to normal cells like neutrophils) and it is the most potent global demethylating agent [39]. Hence, the increase expression of MIEN1 in the immortalized normal epithelial cells, with no similar increase in cancer cells, implies complete demethylation instead of hemimethylation in cancer. The non-nucleoside inhibitors of methylation overcome the toxic effects but are, in general, much less effective than the nucleoside analogues in inhibiting global methylation [40–42]. Procainamide, a specific DNMT1 inhibitor, has been widely studied under this category of drugs [42], primarily due to its existing approval to treat cardiovascular diseases [43]. Although procainamide treatment increased MIEN1 transcript, a more pronounced effect was achieved when silencing all the DNMTs by RNAi. In contrast, the MIEN1 protein levels were not remarkably different between inhibition of only DNMT1 (by procainamide or siDNMT1) or all the DNMTs. This implies the involvement of other factors in RNA and/or protein stabilization when individual DNMTs are targeted.

Interplay between methylation, repeat elements and transcription factor binding are widely responsible for gene transcription [44]. During cancer progression, the presence of SINE repeats in regions near a gene promoter have been shown to enhance gene transcription by directly providing binding access to promoter and enhancer elements which mostly results in oncogenic signaling [45]. The presence of the binding site for USF in the MIEN1 putative promoter, from −18 to −13 relative to the transcription start site, together with the luciferase reporter assays, indicated that USF could regulate MIEN1 transcription. USF is known to bind to E-box consensus sequence CACGTG and result in overall increase in transcription [46, 47, 48]. In support of our finding, previous studies have shown that genes bound by USF have a higher chance of possessing a SINE repeat [5]. Specifically, in terms of Alu elements of the SINE family, there is an equal likelihood of USF binding to a CpG rich or a non-CpG containing promoter [49]. Unmethylated E-box element reduces USF binding which in turn insulates SINE repeat based gene inactivation better than if the site were left unbound [5, 46]. In contrast, our studies revealed that there were no significant differences in the degree of methylation of the E-box between the immortalized normal and the cancer cells (data not shown), implicating involvement of a different mechanism and sequence of events.

It is known that multiple methylation sites and signatures exist at the various gene loci [38]. These sites could either have a clinical correlation or a diagnostic potential with respect to “disease-state” or could just be key in terms of gene regulation or even have the potential to be both. Though this study primarily focused on determining the factors stimulating and inhibiting MIEN1 regulation, we also aimed at identifying any correlation of methylation patterns in and around MIEN1 that might be clinically relevant and support targeting specific methylation instead of global methylation. The TCGA database based methylation studies [50] revealed significant differences in a site other than the site we studied between normal and cancer patients. This shows that determining the probe sets used for such studies play a critical role in understanding and interpreting the results obtained. The regions we studied through bisulfite sequencing were not present within the TCGA probe set and in the cell lines, we did not study the exact same probe sets used by TCGA. Hence, we believe, together, the number of sites studied for methylation differences around MIEN1 is more comprehensive than either one alone making this a robust study encompassing many methylation sites in and around MIEN1. Also, from our results and findings from TCGA, we can conclude that methylation of MIEN1 plays both an important role in the gene regulation as well as diagnosis of cancer, albeit through different sites.

In conclusion, this study is the first to identify methylation as an important modulator of MIEN1 in prostate cancer progression. With our *in vitro* studies, we established that the SINE Alu in the MIEN1 putative promoter region is hypermethylated in normal cells. The methylation of the MIEN1 putative promoter is dependent on both *de novo* as well as maintenance methyltransferases. Loss of this methylation potentially opens the chromatin structure and unveils the MIEN1 promoter for USF mediated transcriptional activation, facilitating the various processes of metastasis. Together, this is an important finding that not only contributes to the knowledge of methylation-based regulation of tumor promoting genes in prostate cancer, but also supports the implications of methylation patterns in disease diagnosis.

## MATERIALS AND METHODS

### Cell lines, cell culture and transfections

Human prostate carcinoma cells LNCaP, DU-145 and PC-3 (ATCC; Manassas, VA, USA) were maintained in RPMI 1640 media supplemented with 10% fetal bovine serum (Life Technologies; Carlsbad, CA, USA). Immortalized non-tumorigenic prostate epithelial cell line PWR-1E was maintained in Keratinocyte-SFM (Life Technologies; Carlsbad, CA, USA), supplemented with bovine pituitary extract (25 μg/ml) and recombinant epidermal growth factor (0.15 ng/ml). All cells were cultured in an incubator at 37°C with 5% CO_2_. The smart pool siRNAs against GFP, DNMT1, DNMT3a and DNMT3b were obtained from Dharmacon (Thermo Fisher Scientific; Waltham, MA, USA) and used at the final concentration of 100 nM. The siRNA transfections for RNA interference were performed using Lipofectamine RNAiMAX according to the manufacturer's protocols (Life Technologies; Carlsbad, CA, USA). Plasmids were transfected using Lipofectamine LTX and Plus reagent according to the manufacturer's instructions (Life Technologies; Carlsbad, CA, USA).

### Genomic DNA isolation, bisulfite modification and sequencing

Genomic DNA from PWR-1E, LNCaP, DU-145 and PC-3 cells was isolated using the Genomic DNA isolation kit (Sigma-Aldrich; St. Louis, MO, USA) according to manufacturer's protocol. Bisulfite treatment and sequencing were carried out at University of Nebraska Medical Center. Bisulfite treatment was carried out using 1000 ng of the genomic DNA and the EZ DNA Methylation-Direct kit (Zymo Research, Irvine, CA, USA), to de-aminate the unmethylated cytosine residues to uracil and leave methylated cytosine residues unchanged. To perform PCR reactions, 32 ng of bisulfite-modified DNA was used as template. The PCR reactions were performed in a total volume of 25 μl for 35 cycles using Roche Diagnostic Corporation's FastStart Taq DNA Polymerase (1.0U), MgCl_2_ solution (3.5 mM), dNTPs (0.2 mM), sense primer (0.24 μM) and antisense primer (0.18 μM) (Supplementary Table S1), with denaturation at 95°C for 30 seconds, annealing for 45 seconds at annealing temperature indicated in Supplementary Table S1, and extension at 72°C for 1 minute. A bisulfite sequencing (BSP) primer set was used to amplify and capture the sequence that contained the region to be analyzed. This was necessary to identify this specific region that contained a repetitive SINE Alu element. This PCR product was used as the template for the internal pyrosequencing primer sets SEQ1, SEQ2 and SEQ3 (Supplementary Table S1). All PCR products were electrophoresed on 0.8% agarose gel, stained with ethidium bromide, and visualized for appropriate and pure product before proceeding with all analyses using a Gel-Doc UV illuminator (Bio-Rad Laboratories; Hercules, CA, USA). Methylation percentage of each CpG was determined using a Pyromark Q24 pyrosequencer (Qiagen; Hilden, Germany, USA) and sequencing primers indicated in Supplementary Table S1, according to manufacturer's recommendations. Bisulfite sequencing was conducted with only one biological sample per cell line.

### Chemicals and treatments

5-Aza-2′dC and Procainamide (Sigma-Aldrich; St. Louis, MO, USA) were dissolved in DMSO and water respectively. The 1mM 5-Aza-2′dC and freshly prepared 1 M Procainamide stock solutions were further diluted in the media (Keratinocyte-SFM or RPMI 1640) to obtain the appropriate final concentrations for treatments. The media with the chemical was carefully added to cells that were seeded one day before and placed in the incubator for the duration of the experiment. Whenever the duration of the treatment was over 48 hours, fresh media with the same concentrations of the chemicals was added to the cells without removing the existing media.

### RNA isolation and qPCR

Total RNA was isolated using TRIzol (Life Technologies; Carlsbad, CA, USA) and quantified. Equal amount of RNA was used for the one-step qPCR performed using the Superscript III SYBR Green qRT-PCR kits, according to manufacturer's instructions (Life Technologies; Carlsbad, CA, USA) on a Mastercycler ep gradient S realplex^2^ thermal cycler (Eppendorf: Hamburg, Germany, USA). The primers were designed using Primer 3 [50] and synthesized by Integrated DNA Technologies (Coralville, IA, USA). The sequences of the primers used are: MIEN1 FP-5′*cagtgctgtggagcagt*3′, MIEN1 RP-5′*gacggctgttggtgatcttt*3′; GAPDH FP-5′*gagcgagatccctccaa*3′, GAPDH RP-5′*actgtggtca tgagtccttc*3′; DNMT1 FP-5′*tacctggacgaccctgacctc*3′, DNMT1 RP-5′*cgttggcatcaaagatggaca*3′; DNMT3a FP-5′*tattgatgagcgcacaagagagc*3′, DNMT3a RP-5′*gggtgttc cagggtaacattgag*3′; DNMT3b FP-5′*ggcaagttctccgagg tctctg*3′, and DNMT3b RP-5′*tggtacatggcttttcgatagga*3′.

### Antibodies and western blotting

The following primary and secondary antibodies were used: Mouse monoclonal MIEN1 (Abnova; Taiwan, China), mouse monoclonal GAPDH (Santa Cruz Biotechnology; Dallas, TX, USA), rabbit DNMT1 and DNMT3a (Cell Signaling Technology; Danvers, MA, USA), and anti-mouse- and anti-rabbit- HRP conjugated IgG (Promega; Madison, WI, USA). Western blotting was performed according to standard protocols. Briefly, once the total protein was isolated using NP-40 lysis buffer, the concentration was estimated using the standard Micro BCA Protein Assay Kit (Pierce Biotechnology, Waltham, Massachussetts, USA). Equal quantity of protein for each sample was run in NuPAGE^®^ Novex^®^ 4–12% Bis-Tris Gels prior to transferring onto nitrocellulose membranes using an iBlot (Life Technologies; Carlsbad, CA, USA). The membranes were blocked in 5% non-fat dry milk and subjected to primary and secondary antibodies before the chemiluminescent reaction was captured by an AlphaImager (ProteinSimple; San Jose, CA, USA).

### Plasmids and luciferase assay

The Firefly-luciferase plasmid, pGL3-basic, a kind gift from Dr. Myoung Kim (UNT Health Science Center, Fort Worth, TX, USA) was used to construct the different reporter plasmids to measure MIEN1 promoter activity. A series of DNA fragments comprising of the nucleotides −581 (KpnI-5′-gcctgaccaacatgatggtaccctgtctctactaaga-3′), −468 (KpnI-5′-cgggaggcagaggtaccagtgaaccgagat-3′), −454 (MluI-5′-gtgaaccgagaacgcgtcattgcactccag-3′), −314 (NheI-5′-catccccaggatgctagccccaccacttt-3′), −215 (KpnI-5′-gaagtccagcgggtaccgggagtgG-3′), −127(NheI-5′-cggggagctagctccggagct-3′) or −3(MluI-5′-tgcccgcggtacgcgtcacac-3′) to +99 (XhoI-5′-cccggctcgagctcctcgggag-3′), relative to the know transcription start site of MIEN1, were PCR amplified and cloned at the KpnI/XhoI or NheI/XhoI or MluI/XhoI sites, upstream of the luciferase gene in pGL3-basic vector. After the sequences were verified (Seqwright; Houston, TX, USA), luciferase assay was performed with the plasmids using the dual luciferase assay kit (Promega; Madison, WI, USA), according to the manufacturer's instructions and luminescence was measured by a Synergy2 Alpha Microplate Reader (BioTek; Winooski, VT, USA). In short, the cells were transiently transfected with the firefly pGL3-constructs and *Renilla* pRL-CMV (a kind gift from Dr. Porunelloor Mathew at UNT Health Science Center, Fort Worth, TX, USA) plasmids. Approximately 72 hours after transfection, cells were lysed and both the firefly and *Renilla* luciferase activities from the extracts were detected. The relative luminescence units (RLU) was calculated as a ratio of firefly to *Renilla* luminescence. The RLU obtained for −314 to +99 pGL3 construct was designated as 100% and the % RLU was correspondingly calculated for the other plasmids. Each construct was transfected at least five independent times and the relative luminescence for each sample was the average of at least three readings.

### Chromatin Immunoprecipitation

Chromatin immunoprecipitation for the USF binding at the MIEN1 promoter was carried out using a ChIP-IT High Sensitivity kit (Active Motif; Carlsbad, CA, USA) according to the manufacturer's protocol. In brief, the DU-145 cells were fixed, lysed and sonicated. After determining the fragmentation and quality of the sonicated chromatin, the immunoprecipitation reaction was carried out with the USF antibodies or the control antibodies. Finally, following reversal of cross linking, PCR was carried out either with the USF binding site specific primers or with non-specific site binding primers and the products were run on an agarose gel. The bands were densitometrically quantified using ImageJ [51] after imaging the gel with an AlphaImager [52].

### Data mining from TCGA

Computational and statistical analyses of TCGA (31) data were performed using R-3.1.2. [53] and Bioconductor 3.1 [54]. These platforms were utilized for integration, preprocessing, quality control assessments, identification of interesting methylation loci, and plotting functionality of publically available Prostate adenocarcinoma (PRAD) Infinium HumanMethylation450 Level 1 data from 479 patients (including tumor matched normal tissue samples from 47 patients). Signal intensities were imported into R using the minfi package [55]. Quality control checks were performed using functions in the shinymethyl package [56] to assess quality control of unmethylated and methylated channels in a large number of samples. Preprocessing and normalization was performed using Subset-quantile within array normalization (SWAN) to correct the technical differences between Type I and Type II array designs [57]. Minfi and shinymethyl were ultimately used to generate relative measurements of methylation signal intensities termed as Beta(β) values and were mathematically converted((M = log2(Beta / (1-Beta))) into a more statistically valid measurement for robust studies termed as M values.

### Statistical analyses

The results were represented as mean ± S.E.M of at least three independent experiments, unless indicated otherwise. The *p-value* was calculated according to Student's *t-test*, or one-way ANOVA with Tukey's Post Hoc test, based on the comparisons made, using GraphPad *P-value* calculator. *P-value* ≤ 0.05 was considered significant.

## SUPPLEMENTARY MATERIALS FIGURES AND TABLES



## Abbreviations

MIEN1: Migration and invasion enhancer 1
SINE Alu: Short interspersed nuclear Alu element
DNMT: DNA methyltransferase
USF: Upstream Stimulatory Factor
BSP: Bisulfite Sequencing
SWAN: Subset-quantile within array normalization
5-Aza-2′dC: 5-Aza-2′-deoxycitidine
PCN: Procainamide
TCGA: The Cancer Genome Atlas.
