# Supplementary material for: MIEN1 is tightly regulated by SINE Alu methylation in its promoter
Source: Oncotarget. 2016 Aug 29;7(40):65307–19. doi: 10.18632/oncotarget.11675 (PMC5323157; doi:10.18632/oncotarget.11675)
Supplement: Supplementary file 1 [file oncotarget-07-65307-s001.pdf]

MIEN1 is tightly regulated by SINE Alu methylation in its promoter

Supplementary Materials

A **-1057**

ttagagttgtaaaatgggaataatgaCGttagtttttagtgagaggttggatgagaagggatgCGtagaaattgttggggtatagagtttgAAGAGTATT
TATTGTTTTTTAGTGTAGGTAAGAAGTAGTTGTAAAAGATTGGAAAAAGTAGTTTAGAGTTTTGGG
ATTAGAAATATTTGTGTATGTAATTATAGGGTGGTTTTAAATGTTATTAGTAtCGggCGtggtggtttaCGttt
gtaatttagtattttgggaggttaaggtaggtagattatttgaggttaggagttCGagattagtttggCGaatatggtgaaattttgttttattaaaataaataaataaat
aaataaaAAATTGggttaggCGCGgtggtttatgtttgtaattttagtattttaggaggtCGaggttggtggattatttgaggttaagagttCGagattagttt
gattaatatgatgaaattttgtttttattaagaatataaaattaatCGggtgCGgtggtaCGCGtttgtagttttagttattCGggaagttgaggtagaagaatCG
tttgaattCGggaggtagaggttgtagtgaatCGagatCGCGttattgtatttagtttgggtaataagagCGaaatttCGttttaaaaaaaaaaaaaaaa
aaTGTTATCGGTATGTTAGCGAGAAAATGTTAATTATTTTATGTATTAGTTATATTTATTTTTATTTTT
AGGATGTTTGTTTTATTATTTTTTTTTTTTTTTGTAAATTGAAAAGCGTTTTGTAGTTTAAGATAGTT
TATTAGAGTAAATGAAGTTATGAAGTTTAGCGGATATCGGGAGTGGGGAGTGGGGAAGTTCGGTA
TTTCGGGAGATCGGGTTAGGGAAGGAGGGTTTGGATCGGATTTAGTTTTTGTTCGGGGAGCGAGT
TTCGGAGTTGTTTTACGAGGTTAAAACGTAGTAGTGGCGGAGATTCGTAGGGGGCGTTCGAACGT
TATTTTCGGTTTTTTTTCGTTTTAGAGGTTTCGTTTCGTTACGTGTTCGCGGTTCGCGTTATATTCG
GAAGTAGGGGTTCGAGCGGAGTCGGTCGCGATGAGCGGGGAGTCGGGGTAGACGTTCGTAGCG
TTTTTTTTCGAGGAGGTCGAGTCGGGTAGTGGGGTTCGTATCGTGGTGGAGTATTGGTGAGCGGTT
TCGGTTGGAGGATTCGTATTTTGGTTTCGCGGGTCGGACGGAGGTGGGTTTACGGGAGGTTTTAT
TTTCGAATTTTTAGTTTAGTTTTATTTTTTGATTTTTTAGTGAATTTTGCGGTTTCGAGGCGATTTAT
TTGGAGTTGGTTAGTGTTGTGAAGGAG **+299**

B

Pre-transcription Start Site Region (SEQ 2)	CpG methylation (> 15 – black; ≥ 10% - grey; < 10% - white)										
**Relative to TSS**	-151	-134	-127	-120	-107	-96	-85	-77	-68	-64	-60
**PWR-1E**											
**LNCaP**											
**DU-145**											
**PC-3**											

C

Translation Start Site Region (SEQ 3)	CpG methylation (> 15 – black; ≥ 10% - grey; < 10% - white)														
**Relative to TSS**	23	27	33	37	39	46	54	63	67	72	82	91	96	110	115
**PWR-1E**															
**LNCaP**															
**DU-145**															
**PC-3**															

Supplementary Figure S1: MIEN1 Promoter. (**A**) MIEN1 methylated promoter sequence. (**B**) Bisulfite sequencing based % methylation at the CpG sites within the pre-transcription start site and (**C**) translation start site regions.

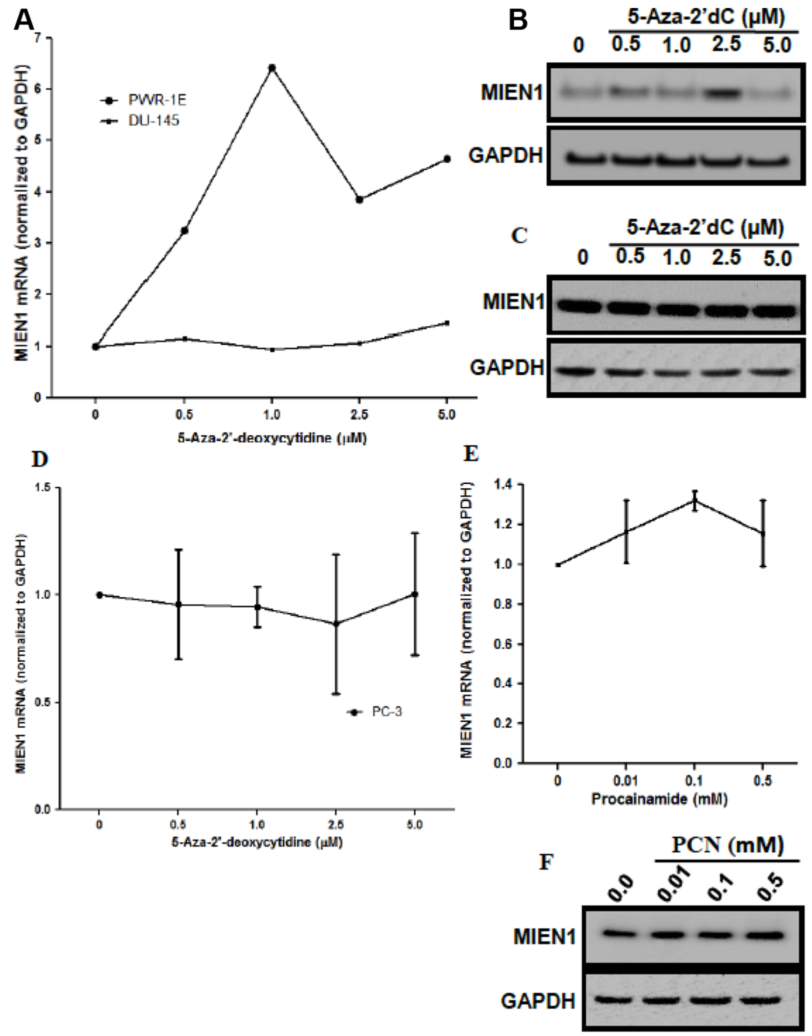

Supplementary Figure S2: MIEN1 expression upon treatment with pharmacological inhibitors. (**A**–**D**) Nucleoside analog, 5-Aza-2'-deoxycitidine treatment: (A) qPCR showing the MIEN1 expression in PWR-1E and DU-145 cells, (B–C) Western Blotting showing the MIEN1 expression in (B) PWR-1E and (C) DU-145 cells, (D) qPCR showing the MIEN1 expression in PC-3 cells. (**E**–**F**) Treatment with the non-nucleoside inhibitor, Procainamide showing MIEN1 expression in PC-3 cells, as depicted by (E) qPCR and (F) Western blotting. GAPDH we used for normalization.

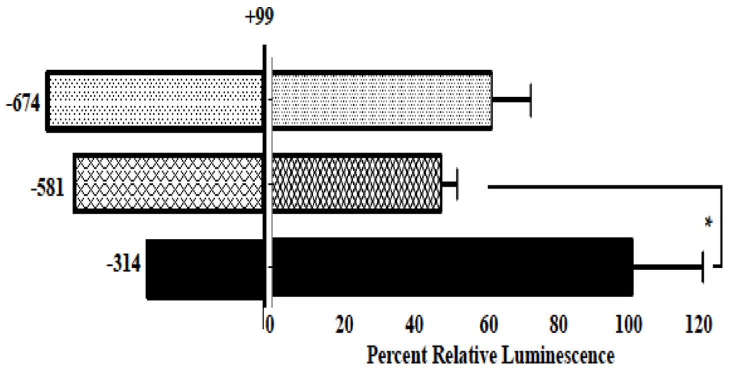

Supplementary Figure S3: Activity of MIEN1 promoter is influenced by SINE Alu. (Left) pGL3 plasmids cloned with the different MIEN1 putative promoter regions. (Right) Promoter activity of different MIEN1 promoter constructs, after transfection in DU-145, read out as relative (Firefly/Renilla ratio) luminescence signals. The P-values were compared using Student's *t*-test between two consecutive constructs. $*P \leq 0.05$.

Supplementary Table S1: Primers for PCR amplification of the promoter regions and for bisulfite sequencing

	Sequence	PCR (bp)	Anneal (°C)
BSP Sense primer (5 ′to 3′)	GGTTGGTGGATTATTTGAGGTTAAGAG	935	63
BSP Anti-sense primer (5′ to 3′)	CTTCACAACACTAACCAACTCCAAATAA		
SEQ1 Sense primer (5′ to 3′)	GAAATTTTGTTTTTATTAAGAATATAA	164	58
SEQ 1 Anti-sense primer (5′ to 3′)	TCTTATTACCCAAACTAAAATACAATA		
SEQ 1 Sequencing Primer (5′ to 3′)	TTTATTAAGAATATAAAATT		
SEQ 2 Sense primer (5′ to 3′)	GGAGATYGGGTTAGGGAAGGAGGGTTT	161	62
SEQ 2 Anti-sense primer (5′ to 3′)	AAACRAAACCTCTAAAACRAAAAAAAAC		
SEQ 2 Sequencing Primer (5′ to 3′)	GGTTAGGGAAGGAGGGTTTG		
SEQ 2 Sense primer (5′ to 3′)	GTTYGYGTTATATTYGGAAGTAGGG	152	62
SEQ 2 Anti-sense primer (5′ to 3′)	CAACCRAAACCRCTCACCAATACTCCAC		
SEQ 2 Sequencing Primer (5′ to 3′)	TTATATTYGGAAGTAGGGG		
